# Measuring Governance: Developing a Novel Metric for Assessing Whether Policy Environments are Conducive for the Development and Implementation of Nutrition Interventions in Nepal

**DOI:** 10.34172/ijhpm.2020.135

**Published:** 2020-08-09

**Authors:** Grace Namirembe, Robin Shrestha, Patrick Webb, Robert Houser, Dale Davis, Kedar Baral, Julieta Mezzano, Shibani Ghosh

**Affiliations:** ^1^Friedman School of Nutrition Science and Policy, Tufts University, Boston MA, USA.; ^2^Department of Community Health Sciences, Patan Academy of Health Sciences, Lalitpur, Nepal.; ^3^Helen Keller International, Patan, Nepal.

**Keywords:** Nutrition Governance, Policy Environments, Metrics, Malnutrition, Nepal

## Abstract

**Background:** The Nutrition Governance Index (NGI) defines a first standardized approach to quantifying the ‘quality of governance’ in relation to national plans of action to accelerate improvements in nutrition. It was created in response to growing demand for evidence-based measures that reveal opportunities and challenges as nutrition-related policies on paper are translated into outcomes on the ground. Numerous past efforts to measure ‘governance,’ most notably World Health Organization’s (WHO’s) NGI and the separate Hunger and Nutrition Commitment Index (HANCI), both of which lack granularity below the national level and each of which fails to capture pinch points related to necessary cross-sectoral actions. This paper addresses such caveats by introducing an innovative metric to assess self-reported practices of, and perceptions held by, administration officials tasked with implementing government policy at the sub-national level. The paper discusses the development of this metric, its methodology, and explores its application in the context of Nepal.

**Methods:** Conducted as part of a nationally representative longitudinal survey across 21 of Nepal’s 75 districts, the sub-study on which this paper is based used data from 520 government and non-government officials at different geographic and administrative tiers of authority. Using robust statistical techniques, structured questionnaire data were condensed into a score using a scale from 0 to 100.

**Results:** Six domains were identified through the analysis: Understanding Nutrition and related responsibilities; Collaboration; Financial Resources; Nutrition Leadership, Capacity, and Support. About half of all health sector representatives achieved a high score (>3 on 5-point scale) compared to representatives in other sectors of government activity (such as agriculture or education) (χ^2^=12.99, *P* <.003). The health sector also showed the most improvement in mean NGI score over a two-year follow-up period.

**Conclusion:** This paper shows that self-reported perceptions and behaviors of those responsible for policy implementation can be usefully quantified. The NGI can be used to assess countries’ readiness for the application of nutrition policies.

## Background

**Key Messages**

** Implications for policy makers**

- With this new metric, policy-makers have access to a tool that measures factors influencing nutrition governance, as reported by those most involved.
- The approach captures the perceptions and practices of professionals from various geographic locations and administrative tiers.
- The tool can be used to quantify achievements and inadequacies in service delivery to provide clearer insight into the effectiveness of nutrition governance, identify points for training to resolve weak performance, and track successes and challenges over time.

** Implications for the public**

With measurable outcomes relating to governance of policy implementation, there is potential for greater transparency and government accountability in service delivery. Because the Nutrition Governance Index (NGI) ranking is simple and intuitive, the public can use this tool to compare governance inputs and outcomes in their locality, compare performance across regions, and enhance local as well as national understanding of the strengths and weaknesses associated with their policy environment thus promoting accountability.

Child undernutrition continues to be a significant global public health concern. Roughly 155 million preschool children were recorded in 2016 as being stunted (too short for their age against internal standards), which represents growth failure at some point in their life from conception through to 5 years of age.^1^ While the trend is downward at a global level, progress in resolving undernutrition is patchy geographically (with Africa and South Asia lagging behind the rest of the world) and too slow. As a result, generations of children in mostly low-income countries continue to face the many health risks, impaired psychosocial development, impeded educational attainment and longer-term economic hurdles associated with malnutrition.^2^

The international community has responded to this challenge by agreeing to a United Nations-endorsed Decade of Action for Nutrition (2016-2025).^3^ Among the targets set for this decade was a 40% reduction in the number of children who are stunted by 2025, to be achieved by an effective consolidation and alignment of international actions and actors.^2,4^ A set of targeted interventions have been agreed, such as accelerating the promotion of exclusive breastfeeding, greater coverage of ante-natal care services, and increasing the access of vulnerable consumers to foods fortified with key vitamins and minerals.^5^

However, while there is broad agreement on many of the technical components of these kinds of programmatic actions for nutrition, it is also widely agreed that successful interventions are ones that are implemented in a conducive or ‘enabling’ policy environment. That is, individual programs are generally cost-effective and sustained if supported by appropriate “structures and policies amenable to project goals.”^6-8^ That is because interventions do not operate in a vacuum; they are implemented in a context that has institutional and individual (human capacity) characteristics that shape how professionals, and non-government stakeholder partners, are able to carry out their responsibilities. Van den Bold et al, identified some important factors including “sensitizing key influencers, political commitment, intersectoral coordination to implement nutrition-relevant policies, adequately resourced nutrition-specific and nutrition-sensitive programs, and sufficient capacities at all levels” as requirements for providing the necessary environment for achieving desired nutrition outcomes in South Asia.^9^

This is where “governance” comes in. The ability of countries to effectively translate policies on paper into desired outcomes on the ground is key to the achievement of national goals. Weak governance has been repeatedly identified as a threat to achieving national and global nutrition goals.^10^ For example, a recent review of 75 studies on the drivers of effective action by governments found that an inability to implement even well-designed policies for nutrition was often linked to “the absence of institutional ownership for nutrition, and institutional failure.”^11^

Unfortunately, the study of governance (successes or failures) has been hampered by the lack of agreed metrics for empirically measuring the processes involved. Most of the literature pertaining to nutrition governance is based in qualitative interviews with key informants and/or desk reviews of the presence/absence of key policy documents and legislation that would be supportive of national nutrition goals.^12,13^ There have been few attempts to establish more quantifiable metrics that could be compared across country situations or monitored over time.^13-15^ In their review of the state of evidence on processes that underpin political and policy successes for nutrition, Gillespie et al concluded that “analyses about how to shape and sustain enabling environments is essential,” and that “the collection of credible metrics … is desperately needed in this area.”^6^

There is a need for a multisectoral approach to ensure delivery of nutrition-specific and sensitive actions to achieve and support nutrition goals.^16^ Although countries have demonstrated strong multisectoral political commitment as documented by release of national multisectoral policies, there is a reported “lack of evidence-based guidance on how to do this, where it makes more sense and how cost-effective such actions would be at scale.”^17^

###  Measuring Nutrition Governance

Defining metrics of governance is a challenge.^7^ Decision-making within and across governments is typically opaque, decisions usually emerge over time rather than appear fully formed as discrete events, and there are many hurdles to “accessing the many different, geographically widespread actors, individuals, groups and networks involved in policy processes.”^18^ As a result, most attempts at standardizing measures of nutrition governance have used national level benchmarks based on available data, such as the presence or absence of certain kinds of policy documents, budgetary allocations, and staffing levels.^19,20^ The 3 recent and widely cited approaches are (*i*) the World Health Organization’s Nutrition Governance Index (WHO’s NGI), (*ii*) the Hunger and Nutrition Commitment Index (HANCI), and (*iii*) the Political Commitment (for Nutrition) Rapid Assessment Tool. Each is reviewed briefly in Table 1.

**Table 1:** Comparing the 3 Recent Metrics of Nutrition Governance

**Characteristics**	**WHO’s NGI**	** HANCI**	**Political Commitment (for Nutrition) Rapid Assessment Tool**
Main focus	WHO ranks governments on their ‘commitment’ (willingness to act) and ‘capacity’ (readiness to act).^19^	Rank governments on their political commitment to tackling undernutrition while seeking to measure what governments achieve and where they fail.	Designed to offer deeper insight into individual countries’ depth of political commitment to food security and nutrition.^21^
Definitions	Capacity was determined in relation to the skills, knowledge, satisfaction and motivation, accountability and freedom of action of individual professionals (staff) within responsible organizations.	10 indicators were related to commitment to hunger reduction and 12 indicators relating to commitment to address undernutrition.	Expressed verbal commitment by high-level, influential political leaders, institutional commitment and budgetary commitment.
Rationale for creating the index	Parameters provide salient insight into the nature of organizational entities and the characteristics of individuals tasked with delivering improved nutrition at country level. Lack of appropriate measures or indicators that would help understand (*a*) roles and responsibilities of individuals and organizations, (*b*) the capacity and areas of competence required of the responsible workforce, and (*c*) metrics of process (not just outcomes) to allow for improved nutrition governance.^22^	To shine a spotlight on what governments are doing or failing to do in their commitment to end hunger and undernutrition.	“Political commitment for food and nutrition is rarely adequately defined or empirically measured.” “Low political commitment has been recognized as a barrier to the scale-up of proven effective food and nutrition policies.”
How indicators are assessed	Assessed in terms of measures of input such as political. Acknowledgement of the problem, the existence of relevant policies, and resource mobilization at central level (along with budgetary alignment at sub-national level).	In terms of measures of ‘input’ such as spending on nutrition rather than on ‘outcomes’ such as levels of stunting.	In terms of measures of input relating to organizational structures, funding for programs, and the legal and regulatory environment.
Number of countries ranked	Ranks 36 low/middle-income countries with the highest burden of child stunting, using 11 indicators.	Ranks 45 countries using 22 indicators grouped under 3 themes: public expenditure, policies and programs, and legislative agendas.	Ranks 10 low-income countries.
Methodology of the index	Indicators are equally weighted. The index ranges from 0 to 11.^23^ The “strength of nutrition governance” was classified as ‘weak’ for countries scoring from 0 to 6.9, ‘medium’ for those scoring from 7 to 9.9, and ‘strong’ for those scoring from 10 to 11.0.	Hunger and nutrition are treated as equally important.^21^ All 3 themes are weighted equally.	Some questions are yes/no and other questions allow for a range of relative responses from 1 through 10. For the latter questions (on a scale), a response of 7 or higher was allocated a 1, and for questions relating to budgets (with a 0 to 3), a response of 3 was assigned a 1. The total score possible is 51.
Data sources	GINA	Various databases such as IFPRI (SPEED database), WHO Global Health Observatory Data Repository, IFAD, SUN Compendium of fiches, etc.	Theory-based survey based on existing literature of political commitment and “questions permitting a rapid stakeholder analysis to assess the positions and power of major country-level actors in food and nutrition."
Key country outcomes	Strong nutrition governance: Peru, Malawi, and Vietnam. Weak nutrition governance: Cambodia, Mali and Pakistan, Afghanistan, Iraq, Yemen, and DRC.^23^	Greatest commitment to nutrition: Peru, Malawi and Guatemala. Very low commitment: Cambodia, Pakistan, Afghanistan, Yemen, and DRC.^24^	Highest ranked: Philippines and Colombia. Lowest ranked: Vietnam and Bangladesh ranking.^21^
Example of a study that has referenced the metric	Harris et al^25^	te Lintelo et al^26^	Li et al^27^
Year(s) study was conducted	2007-2008	2012-2013	2016
Advantages	Ranking of countries is intuitive.	Offers insight into “the general quality of public administration in a country.”	Allows for discrimination among the various elements that make up the final score, making it more useful to any analysis of the elements of governance that are stronger or weaker.
Limitations	Lack of granularity at sub-national level (inability to differentiate across sectors of government activity or below the national level of government). Unable to determine which facets of governance appear to be more, or less, related to policy-driven actions. Being based on official data collated at national level there is limited change across years.	Lack of granularity at sub-national level. Offers little specific to nutrition or to the quality of the process of implementation of policies where they exist.^20^	Still relies mainly on an assessment of information that exists at national level that may or may not relate directly to a government’s ability to implement pro-nutrition policies and programs.

Abbreviations: NGI, Nutrition Governance Index; HANCI, Hunger and Nutrition Commitment Index; WHO, World Health Organization; GINA, Global database on the implementation of nutrition action; IFAD, The International Fund for Agricultural Development; DRC, Democratic Republic of the Congo; SPEED, Statistics on Public Expenditures for Economic Development; IFPRI, International Food Policy Research Institute.

The alternative approach proposed in this paper focuses on an empirical compilation of information derived through in-person surveys with professionals and other stakeholders holding positions and responsibilities for achieving nationally defined nutrition goals. We based our selection of survey questions on published literature about the key factors that proved to be relevant to nutrition governance, some of which have been mentioned in Gillespie et al. They identified 3 domains; knowledge and evidence, politics and governance, and capacity and resources as essential factors for good governance.^7^ Another important factor studied was support or commitment from stakeholders.^28^ In this sense, the nutrition governance score is more directly based on what people know, think and do in relation to their defined areas of responsibility at all levels of administration, across all sectors.

This paper introduces a novel metric for assessing ‘nutrition governance.’ Using empirical data collected in Nepal, we used a principal component analysis (PCA) approach to calculate an NGI based on participants’ weighted responses on major facets of governance drawn from the literature. We validated the tool using a confirmatory factor analysis (CFA).^29^ The goal of this work is to develop a quantifiable metric that informs whether a local policy environment is likely to be enabling or constraining for the development and implementation of nutrition interventions based on the perceptions of relevant stakeholders involved in nutrition governance.

## Methods

###  Sample Size and Study Design

This study was conducted as part of the Policy and Science for Health, Agriculture and Nutrition (PoSHAN) study (detailed methods are described in Klemm et al).^30^ The latter was a nationally and agro-ecologically representative panel study that was conducted annually from 2013 to 2016. Over 5000 women and children were sampled across 21 village development committees (VDCs), each located in a separate district. Three wards per VDC were selected (n = 63 wards) and all eligible households with a child under 5 years were recruited. Each survey provides a wealth of data on markets, community, household and individual factors that are associated with nutrition outcomes.^31^

The Nutrition Governance Study followed the same sampling design and timeframe as the PoSHAN study, except that the governance study interviewed office holders and organizations within the VDCs rather than men and women within households. Offices were chosen based on their defined responsibilities in implementing Nepal’s Multisector Nutrition Plan (MSNP).^17^ The MSNP is a collaborative multi-national partnership spearheaded by the government of Nepal to improve maternal and child nutrition and reduce chronic malnutrition, largely through evidence-based nutrition interventions. These tasks are overseen and executed primarily by five ministries; Ministry of Agriculture Development, Ministry of Health and Population, Ministry of Education, Ministry of Urban Development and the Ministry of Federal affairs and Local Development.^32^ Two districts, Jumla and Nawalparasi, were in the MSNP catchment area at the time of data collection.

 This study used the 2016 survey round, which included a total of 520 government and non-government officials from these ministries, spanning four managerial levels (District, Ilaka, VDC, and Ward). The district is the highest managerial level concerned with executive planning, budgeting and facilitating multiple nutrition-related activities. The next hierarchical level was (at the time of the survey) the Ilaka, which further facilitated nutrition-related activities. The VDC level has many government representatives to serve and engage with the community; at the ward level (there are roughly nine wards in each VDC), one finds frontline workers interacting with individual farmers, healthcare seekers, etc.

The questionnaire comprised a set of 24 multiple interrelated Likert-scale items/questions that were used to gather data on self-perceived governing practices within respondents’ scope of roles and responsibilities (See Supplementary file 1). Each participant answered one of four options: “Strongly Agree,” “Agree,” “Disagree,” and “Strongly disagree” and for some questions, “Don’t Know” or “Not Applicable.” The scale was reconstructed to “Strongly Agree,” “Agree” and “Disagree,” the latter indicating the respondent selected either “Strongly disagree” or “Disagree.”We attributed an arbitrary penalty of 0.25 to “Agree” responses in order to account for the loss of certainty. This ensured that these responses carried less weight in the PCA procedure compared to “Strongly agree” responses. The lesser degree in agreement was represented in the factor scores created resulting in more robust principal components. A larger penalty (0.5) would insinuate that a slight shift from strong agreement reduces the positive effect by half – an assumption too strong to make.

 The same questionnaire was used for each respondent regardless of management roles and administrative level. Whereas respondents differed in their responsibilities, the items were broad enough to be relevant across administrative levels yet specific enough to apply to singular roles.

 Thirty eight percent of all the participants were from the district level, 12% from the Ilaka level, 16% from the VDC and 34% from the Ward level. 56% were government representatives with the rest from various non-governmental offices. Disaggregated by management level, 59% of all the government officials were from the district, 21% from the Ilaka level and 21% from the VDC level. Majority of participants from non-governmental offices came from the Ward level (82%). Government officials had been in their current positions for a median duration of 2 years compared to 3 years for non-government officials. Twenty-eight percent of all the participants had acquired a graduate degree, 16% had an undergraduate degree, 49% had less than a college degree while 7% had no formal education.

Table 2 shows the percentage distribution of participants’ responses across the 4 management levels.

**Table 2:** Percentage Distribution of Responses at the 4 Management Levels

**Item**	**District (n = 198)**			**Ilaka (n = 62)**			**VDC ( n = 81)**			**Ward (n = 179)**		
	**0** ^a^	**0.75** ^b^	**1** ^c^	**0**	**0.75**	**1**	**0**	**0.75**	**1**	**0**	**0.75**	**1**
1	09	79	12	02	87	11	10	81	09	06	82	12
2	17	69	14	16	77	06	20	74	06	20	69	12
3	21	62	16	19	71	08	31	57	09	34	53	02
4	26	61	12	18	71	11	33	51	16	27	60	12
5	13	75	12	05	77	18	07	84	09	10	76	14
6	18	68	15	10	77	13	20	73	07	17	66	15
7	33	60	07	13	76	10	21	74	05	17	73	09
8	02	45	53	02	18	81	00	42	58	01	49	51
9	22	75	04	35	55	06	26	70	02	35	56	07
10	22	67	11	18	69	11	16	78	06	16	77	06
11	13	75	12	13	71	16	12	75	12	19	69	12
12	27	69	03	39	55	05	30	69	01	28	64	03
13	17	74	09	39	58	02	31	68	01	26	66	07
14	29	66	03	34	60	02	31	63	05	35	54	04
15	21	67	13	35	63	02	47	48	05	64	28	01
16	38	55	07	65	26	10	60	36	04	69	22	01
17	64	32	04	45	50	05	75	23	01	58	35	03
18	39	54	08	21	73	05	42	54	02	33	60	06
19	35	52	13	39	53	08	43	49	07	44	46	10
20	43	54	03	40	52	08	41	51	09	44	51	04
21	03	56	42	00	63	37	02	63	35	02	79	20
22	03	74	23	00	73	27	09	72	20	04	74	22
23	01	78	21	00	73	27	01	78	21	02	83	15
24	07	81	12	06	85	08	11	83	06	10	84	06

Abbreviation: VDC, village development committee. ^a^0 = Disagree, ^b^0.75 = Agree, ^c^1 = Strongly agree.

###  Statistical Analysis

We approached the identification of ‘domains of governance’ using PCA.^33^ PCA is a data reduction procedure for transforming a large number of variables into a smaller number of uncorrelated (orthogonal) factors, the principal components. These factors account for some of the variation in the data. They are ordered in such a way that the first component captures the most variation. Subsequent components are completely uncorrelated and account for the maximum variation that is not previously accounted for. Items that loaded on a component were assessed for meaning and interpretation. Similar items that load on a retained component attribute meaning to a domain. After a second round of PCA on each identified domain, only one component was retained, which confirms the unidimensional nature of the identified domains.

Factor scores were calculated to indicate each participant’s position on the retained components. The observed items were standardized to a mean of 0 and a variance of 1. These standardized variables are then multiplied by their respective standardized scoring coefficients. The products are then summed over all the variables per domain and the sum is the value of the factor score. This was calculated in SAS using the SCORE function in the PROC FACTOR procedure. Because the retained components account for varying levels of the total variance in the data, we accounted for this difference in their importance by using equation 1^34^:


(1) $$H_{j}=\sum S^{2}{}_{kj}f_{jk}\;j=1,2,...,j$$

The percentage for the kth factor is denoted by

$$S^{2}{}_{k}$$

and *j* represents each individual. For example, for each individual, the unstandardized, weighted NGI is calculated as:

####  NGI = ((47 x Knowledge factor score)+(54 x Collaboration factor score) +(75 x Financial Resources factor score)+(74 x Leadership factor score) +(72 x Capacity factor score)+(41 x Support factor score)) 

where the values 47, 54, 75, 74, 72 and 41 are the percent variances for each domain. The unstandardized index ranged from -758 to 545, which was difficult to interpret so we standardized it using the min-max normalization formula in equation 2 below:


$$I_{i}=\frac{\left(H_{j}-H_{\min imum}\right)}{\left(H_{\max imum}-H_{\min imum}\right)}\;j=1,2,...,j$$

The standardized NGI score ranges from 0 to 100, with higher scores indicating better nutrition governance.

###  Validating the Nutrition Governance Index

Cronbach’s alpha coefficient was calculated to determine reliability of the NGI. Reliability tests assume that the unidimensional assumption holds, therefore these tests were conducted by their domains as opposed to across all domains. Reliability describes the extent to which all items in a test measure the same underlying trait.^35^ These estimates range from -1 to +1, with higher absolute values indicating higher reliability. It is also a test for measurement error: as reliability increases, measurement error decreases. A criterion of 0.7 and above is generally considered acceptable.^36^ Low alpha values imply that the domains require reassessment to either increase the number of items or replace them altogether.

The construct validity for the NGI was examined using CFA.^29^ This technique was used to verify the factor structure imposed on the data, that is, whether the domains obtained were a good fit. The ‘goodness of fit’ test was developed as an alternative to the chi-square test. It calculates the proportion of variance accounted for by the estimated population covariance.

The root mean square error of approximation (RMSEA) is a calculation based on the χ^2^ and sample size. It tells us how well the model, with unknown but optimally chosen parameter estimates would fit the population’s covariance matrix.^37^ A value of 0 indicates perfect fit^38^ with a recommended value of ≤0.06 as a cut-off for good fit. Similar to the RMSEA, a standardized root mean square residual (SRMR) value of 0 indicates perfect fit and a cut-off of ≤0.08 is indicative of a good fit.

The null model for Bentler’s comparative fit index assumes that all latent variables are uncorrelated and compares this model to the sample covariance matrix. Values range from 0 to 1.0, with values closer to 1.0 indicating a good fit. A value ≥0.95 is recognized as the cut-off for a good fit.^37^

## Results

###  Nutrition Governance Index Domains

Table 3 shows the domains that emerged from the analysis of the survey data. Six domains were identified using PCA; Understanding Nutrition and responsibilities, Collaboration, Financial Resources, Nutrition Leadership, Capacity, and Support.

**Table 3:** Items Under Each Domain and the Corresponding Reliability Coefficient for Each Domain

**Item**	**Domain**	**Cronbach's Alpha**
	**Domain 1: Understanding Nutrition and Their Responsibilities**	**0.83**
1	Adequate understanding of nutrition problems to be able to implement strategies	
2	Sufficient discussion among office colleagues on how to implement strategies	
3	Nutrition is taken formally into consideration in annual plans and budgets	
4	Personal responsibilities related to nutrition are clearly defined	
5	They know when own actions have been successful or effective	
6	Work-related decisions are based on hard data/technical evidence in nutrition	
7	Most colleagues consider nutrition a priority for them to work on	
8	Improving nutrition is one of the responsibilities of their sector	
	**Domain 2: Collaboration**	**0.83**
9	There is effective collaboration across offices in addressing nutrition issues	
10	Supervisors actively promote collaboration with other offices	
11	There is sufficient sharing of information about nutrition plans and activities	
12	Necessary stakeholders are included in discussions to address nutrition issues	
13	There was demand from other ministries for their offices to collaborate with them	
14	They effectively collaborated with their coworkers to address nutrition problems	
	**Domain 3: Financial Resources**	**0.68**
15	They personally have sufficient access to budgetary resources to be effective in their roles	
16	Their office has sufficient financial resources to implement actions to meet their roles	
	**Domain 4: Nutrition Leadership**	**0.65**
17	They have a champion for nutrition in their sector	
18	There is clear leadership on nutrition in their sector	
	**Domain 5: Capacity **	**0.60**
19	They have personally been adequately trained to carry out their responsibilities	
20	Their own colleagues have the right skills or training to be effective in their work	
	**Domain 6: Support**	**0.51**
21	They know how to obtain any technical support for their responsibilities	
22	They have adequate support from their supervisors for implementing their roles	
23	They have adequate commitment from colleagues in their sector to help fulfill their roles	
24	Sufficient non-financial resources are made available	

Applying the criteria of the Cronbach’s alpha coefficient test for reliability, only two domains (1 and 2) were reliable, domains 3 and 4 were borderline reliable, 5 and 6 were unreliable (Table 3).

Table 4 shows the NGI’s goodness of fit indices using a CFA technique to examine construct validity.

**Table 4:** Goodness of Fit Statistics for Confirmatory Factor Analysis

**Fit Summary**	**Goodness of Fit Measure**	**Fit Estimate**	**Cut-off Limit**	**Pass or Fail**
Absolute Index	Fit function	1.09		
	Chi-square	488.96		
	Pr > Chi-square	<0.00		Fail
	SRMR	0.05	<0.08	Pass
	Goodness of fit index	0.92	>0.90	Pass
Parsimony index	Adjusted goodness of fit index	0.89	>0.90	Pass
	RMSEA estimate	0.05	<0.06	Pass
Incremental index	Bentler comparative fit index	0.92	≥0.95	Fail

Abbreviations: SRMR, standardized root mean square residual; RMSEA, root mean square error of approximation.

 Overall, the domains obtained are a good fit for the data. Whereas the model did not pass the chi-square and Bentler’s comparative fit index, it passed all the other indices thus confirming the factor structure imposed. Nevertheless, based on relatively poor reliability for some domains and failure to meet some fit indices, we recommend re-assessing the items in domains with low alpha values to improve overall model fit.

###  Ranking Sub-national sectors across time

 Figure summarizes the performance of various groups based on their median NGI score in 2014 and 2016. The gap between the two time points intuitively reveals the extent of improvement across time; the larger the distance to the right between points of the same group, the greater the improvement in governance.

**Figure:**
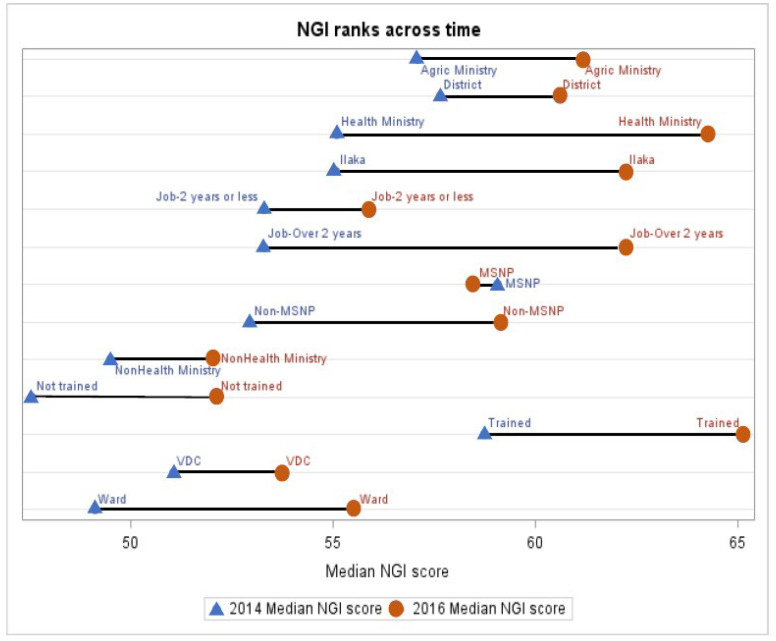
Median NGI Score by Respondent Groups. Abbreviations: NGI, Nutrition Governance Index; VDC, village development committee; MSNP, Multisector Nutrition Plan.

Table 5 shows the percentage distribution of respondents categorized by NGI ranks. The NGI ranks are a quintile distribution of the NGI score, that is, the NGI was categorized into 5 equal parts from lowest to highest score. The NGI scores were computed using the 2016 survey data.

**Table 5:** Percentage Distribution of Respondent Groups by the NGI Ranks/Quintiles From Lowest (1) to Highest (5)

	**NGI Ranks/Quintiles (N = 520)**				
	**1**	**2**	**3**	**4**	**5**
Ministries					
Agriculture	13.64	20.45	20.45	26.14	19.32
Health	12.68	15.49	22.54	23.94	25.35
Non-Health	29.68	24.20	17.35	13.70	15.07
Level of management					
District	12.12	22.22	19.19	22.73	23.74
Ilaka	14.52	9.68	32.26	22.58	20.97
VDC	25.93	23.46	17.28	14.81	18.52
Ward	27.93	19.55	17.88	18.44	16.20
MSNP membership					
No	19.87	20.09	19.02	20.09	20.94
Yes	21.15	19.23	28.85	19.23	11.54
Nutrition training/courses received					
No	29.75	21.86	18.28	15.41	14.70
Yes	8.71	17.84	21.99	25.31	26.14
Length in current position					
Less than 1 month	00.00	50.00	50.00	00.00	00.00
2 years or less	22.96	21.40	19.84	15.18	20.62
Over 2 years	17.24	18.39	19.92	24.90	19.54
Level of education					
Bachelors	16.05	19.75	23.46	20.99	19.75
Intermediate (10 + 2)	24.05	20.25	21.52	18.99	15.19
Just literate (non-formal education)	21.05	23.68	18.42	23.68	13.16
Masters	11.19	22.38	21.68	20.98	23.78
PhD	00.00	00.00	100.00	00.00	00.00
Primary (up to grade 5)	29.73	18.92	16.22	18.92	16.22
Secondary (grade 6-10)	31.73	19.23	15.38	15.38	18.27
Technical degree	10.81	10.81	18.92	27.03	32.43

Abbreviations: NGI, Nutrition Governance Index; VDC, village development committee; MSNP, Multisector Nutrition Plan.

Based on Table 5, we aggregated the highest quintiles (4 and 5) into one category to create a binary variable of highest vs. lowest NGI scores and run tests of association with some of the categories in the table. The results are presented below.

###  Ranking Ministries/Sectors

The health sector showed the most improvement in mean NGI score followed by the Agriculture sector. About half of the respondents from the health sector were associated with achieving a higher score (4 or 5) compared to other sectors (χ^2^ = 12.99, *P*< .003). This is an important finding because the Health sector is at the forefront of all health-related and some agriculturally relevant activities including operationalizing and advocating for maternal and child nutrition programs.

###  Rating the Nepal Government’s Multi-Sector Nutrition Plan 

Only a third of respondents exposed to MSNP initiatives were ranked in the highest scoring categories (4 and 5) compared to 41% not exposed (χ^2^= 2.05, *P* = .152). Furthermore, across time, the mean NGI score was lower for respondents from these districts. This could be explained by the fact that there were only 2 out of 21 districts from the MSNP catchment area at the time of data collection. Nevertheless, this finding serves (1) to show MSNP policy-makers that there are areas of improvement to be explored and (2) to avail concerned parties a tool against which they can measure MSNP performance.

###  Ranking Staff Trained in Nutrition Related Activities

Building local capacity by putting an emphasis on attending nutrition courses and training improved nutrition governance. Half of the respondents who received nutrition training were associated with achieving a high score compared to those who had no training (χ^2^= 24.55, *P* <.000). Training at the implementation level raises more awareness of the nutrition issues and increases the likelihood of understanding the effectiveness of intersectoral and cross-sectoral coordination. Similarly, the more extensive the work experience, the higher the likelihood of performing better on the job and the greater the score on the NGI (χ^2^= 4.31, *P* < .038).

## Discussion

The findings presented here support the use of a standardized approach to measuring ‘governance’ through self-reported behaviors and opinions of office holders responsible for implementing policies at various tiers of administrative responsibility. The index developed for this purpose offers valuable insights into a wide range of issues that are relevant to the successful or unsuccessful implementation of government agendas. Similar to other studies,^6,23,28^ the domains that make up the composite index were identified based on their relevance in determining the status of the nation’s nutrition policy environment. An index of this kind has relevance not just at the national, but at multiple sub-national levels where such policies must be implemented. This study has also shown that such an index is relevant to the work of all pertinent ministries, in many different geographic contexts, which suggests its potential for wide replicability, both beyond Nepal and for assessing governance in relation to issues other than nutrition.

The NGI’s ability to assess different facets of governance, and to identify which appears to be enabling versus impeding success in the policy environment makes this metric stand out as having important real-world potential. Being able to test this approach on a multi-year basis also permitted an assessment of the tool’s sensitivity to potential changes in the short-run – a feature that is important to planners who seek to understand how well or poorly activities are being undertaken in real time. For example, it was possible to detect a change (a large positive shift) in the median NGI among respondents who work in the health sector compared with other sectoral responsibilities, likely as the result of increased training and defined responsibilities for health sector workers in the early years of roll-out of the government’s national nutrition strategy. This is suggested by the higher shift in NGI among those individuals reporting that they had received nutrition and health training, as well as by those who had more years of experience in the job and could more easily access appropriate information and resources to undertake required work. More trainings seems to have resulted in better understanding of nutrition issues, coupled with a higher likelihood of seeking to work in a coordinated fashion across and within different sectors to solve nutrition challenges, in line with what we previously found.^17^ The ability of the NGI to single out where positive change and remaining challenges lie (by sector, geography and administrative tier) offers genuine value to governments seeking to define where investments (such as training, resource allocation or other capacity building) should be prioritized or reallocated.

 The 6 domains explored through the NGI represent the key areas offering challenges and potential solutions as reported by those most involved in policy implementation. In Nepal, the NGI identified successes and continued weaknesses across sectors and tiers of governance relating to how individuals understand the causes of nutrition problems, perceptions of office-specific roles, responsibilities in tackling those problems, capacity and areas of technical competence relating to the implementation of solutions, leadership, support in day-to-day functioning from professional managers and collaboration with peers, and appropriate access to budgets.

Two of these domains, understanding nutrition problems, on the one hand, and clear definition of roles and responsibilities, on the other hand, emerged as highly statistically reliable domains of governance. Thus, the NGI goes beyond previous governance metrics^19-21^ by empirically exploring metrics of process (related to policy implementation) rather than just inputs or outcomes long after the fact. Box 1 provides a summary of the characteristics of the NGI.

**Box 1. Summary of the Relevant Characteristics of the NGI**

- Measures the constituent parts of ‘governance’ index at the sub-national level, across sectors, and over time.
- Measures different domains of governance, including some that have not been explored in previously developed indexes.
- Uses robust statistical techniques for validation and sensitivity analysis.
- Based on a structured questionnaire survey that elicits self-reported data on what people know and do; this standardized approach allows for comparison across locations, tiers of governance and context.
- While the domains of interest are not equally weighted, the variability in responses for each domain is accounted for.
- It is a relatively simple and intuitive tool, easy to interpret by policy-makers as well as by civil society activists at local level who seek greater government accountability.

Abbreviation: NGI, Nutrition Governance Index.

An analysis of response patterns in Table 2, showed that there was agreement across all administrative tiers with the exception of Item 15, which inquired about access to budgetary resources. The disproportionately low percentage of respondents who strongly disagreed at the lower levels of management could be explained in two ways; (1) Their roles do not involve handling budgets so the question is irrelevant; (2) They genuinely strongly disagree with the item. All the 13 respondents who opted for either the “Don’t Know” or “Not applicable” categories were from the Ward level which points us to the first explanation. A similar analysis of missing value patterns showed that Item 3 and Item 16 may not be applicable to lower levels of management, therefore rating them using these items reflects an unfair sense of judgement in their overall NGI score.

###  Limitations and Future Research Priorities

 This novel index assesses nutrition governance at the sub-national level, which is of great potential value to both researchers and policy-makers keen on gaining an improved understanding of complex multi-institutional policy environments. However, a high score on the NGI may not necessarily mean a high score on each of the domains, so sub-national levels should be reviewed by NGI domain in order to gain better insight into the perception of governance practices.

 The sampling strategy used for these analyses was purposive in design. Although effective in achieving meaningful results (when well executed), this strategy can be problematic when making inferences. Inferential statistics allow for generalization of results to a much larger population using sampled data. Whereas we sampled from across the 3 major agro-ecological regions in Nepal, participants were sampled non-probabilistically on a smaller scale within each zone thus there would have been a selection bias if perceptions of governing practices captured in this study differ from participants’ not included in the study. Consequently, the results obtained in this study may not be applicable nationwide or on a much greater scope due to limited representation.

 The process of item selection was iterative in practice. The first round of data collection was qualitative because we took an exploratory approach that allowed us to gain a broad understanding of governing practices on the ground. At each subsequent round, items were refined using PCA, which resulted in replacing uncorrelated items with new, relevant items under each domain. We used the fourth round of data collection in this paper, to construct the nutrition governance indicator which poses the following limitations; (1) some of the items were not asked in previous rounds therefore the outcome of the NGI is dependent on the round of data collection; (2) it may be problematic to compare trends in NGI across rounds.

 As aforementioned, the first principal component accounts for the most variation but it explains only a small proportion of all the variation under each domain. Although all the items under each domain fell on a single component, any new items added may not. The use of only one component is therefore restricting as it omits a great wealth of information in the final index and can imply unidimensionality where it may not exist. However, the aim was to obtain a single index representing each domain therefore this approach was necessary. In addition, since we only considered the factor with the highest variation, there will not be significant benefit in adding higher-order factors as they tend to explain much smaller proportions of variation.

## Conclusion

 This paper demonstrates that various important facets of ‘governance,’ associated in this case with implementation of a nutrition strategy, can be quantitatively measured in ways that offer insight into strengths and weaknesses within policy environments. It lays an important groundwork for future studies that aim to measure governance in other sectors and spheres of action. Most of the published indices have a focus on measurements at a national level, but the NGI is one of a few that can measure perceptions and practices relevant to policy implementation at a sub-national level. This tool can be used to quantify achievements and inadequacies in service delivery to provide clearer insight into the effectiveness of nutrition governance, guide policy-making and track performance over time.

 Future research ought to focus on validating this tool in different settings or countries to assess its generalizability. We also recommend conducting studies that determine the relationship between nutrition governance and nutrition outcomes. This will shed light on how policies can translate into improved nutrition outcomes.

## Acknowledgements

 Special thanks go to the POSHAN data collection team and participants for their time and collaboration. We are particularly grateful to the Nutrition Innovation Lab team for their support and input over the course of several meetings, in which issues presented in this paper were zealously discussed.

## Ethical issues

 The study was approved by Tufts Social Behavioral and Educational Research Board (SBER) and Nepal Health Research Council (NHRC).

## Competing interests

 Authors declare that they have no competing interests.

## Authors’ contributions

 PW, SG, DD, KB, and RS were the primary contributors to the conception and design. PW, GN, JM, and RS contributed to the writing and editing of the manuscript, GN performed the statistical analyses and consulted with RH. PW, RH, SG, and RS provided important intellectual content and interpretation of results.

## Authors’ affiliations

^1^Friedman School of Nutrition Science and Policy, Tufts University, Boston MA, USA. ^2^Department of Community Health Sciences, Patan Academy of Health Sciences, Lalitpur, Nepal. ^3^Helen Keller International, Patan, Nepal.

## Funding

 This work was supported by the United States Agency for International Development (USAID) under grant number [AID-OAA-L-10-00006]. The funder did not have influence on the outcome or conduct of this study.

## Supplementary files


Supplementary file 1. Nepal PoSHAN Policy Process Research R4 (2016).
