# Supplementary material for: Measuring Governance: Developing a Novel Metric for Assessing Whether Policy Environments are Conducive for the Development and Implementation of Nutrition Interventions in Nepal
Source: Int J Health Policy Manag. 2020 Aug 9;11(3):362–73. doi: 10.34172/ijhpm.2020.135 (PMC9278471; doi:10.34172/ijhpm.2020.135)
Supplement: Supplementary file 1 — Nepal PoSHAN Policy Process Research R4 (2016). [file ijhpm-11-362-s001.pdf]

Supplementary file 1. Nepal PoSHAN Policy Process Research R4 (2016)

	**Explaining the Format of the Interview**
	I will start each question with the following words: "Do you feel that in the past 12 months…" then make a statement. You will then have the chance to strongly agree/agree/ disagree/ strongly disagree with that statement. (Enumerator note: If the official has been in the position for less than 12 months please ask to their best knowledge since they joined the position)
**No.**	**Question**
1.	Do you feel that you yourself have an adequate understanding of nutrition problems to be able to effectively implement the strategy in this sector?<br>1. Strongly Agree<br>2. Agree<br>3. Disagree<br>4. Strongly disagree<br>98. Don't know
2.	Do you feel that there is sufficient discussion among your own office colleagues on how to implement the strategy?<br>1. Strongly Agree<br>2. Agree<br>3. Disagree<br>4. Strongly disagree<br>98. Don't know
3.	Do you feel that nutrition is taken formally into consideration when your sector is preparing its annual plans and budgets?<br>1. Strongly Agree<br>2. Agree<br>3. Disagree<br>4. Strongly disagree<br>98. Don't know<br>99. Not applicable

4.	Do you feel that your own (personal) responsibilities in relation to actions on nutrition are clearly defined?<br>1. Strongly Agree<br>2. Agree<br>3. Disagree<br>4. Strongly disagree<br>98. Don't know<br>99. Not applicable
5.	Do you feel that you know when your own actions have been successful or effective?<br>1. Strongly Agree<br>2. Agree<br>3. Disagree<br>4. Strongly disagree<br>98. Don't know
6.	Do you feel that your work related decisions are based on hard data or technical evidence relating to nutrition?<br>1. Strongly Agree<br>2. Agree<br>3. Disagree<br>4. Strongly disagree<br>98. Don't know
7.	Do you feel that most colleagues (in your office/department) consider nutrition as a priority for them to work on?<br>1. Strongly Agree<br>2. Agree<br>3. Disagree<br>4. Strongly disagree<br>98. Don't know
8.	Do you believe that improving nutrition is one of the responsibilities of your sector?<br>1. Strongly Agree<br>2. Agree<br>3. Disagree<br>4. Strongly disagree

	98. Don’t know
9.	Do you feel that there is effective collaboration across offices/ministries in addressing nutrition issues?<br>1. Strongly Agree<br>2. Agree<br>3. Disagree<br>4. Strongly disagree<br>98. Don’t know
10.	Does your supervisor/manager actively promote collaboration with other offices/sectors?<br>1. Strongly Agree<br>2. Agree<br>3. Disagree<br>4. Strongly disagree<br>98. Don’t know
11.	Do you feel that there is sufficient sharing of information about the nutrition plans and activities of other offices/sectors?<br>1. Strongly Agree<br>2. Agree<br>3. Disagree<br>4. Strongly disagree<br>98. Don’t know
12.	Do you feel that all necessary stakeholders are included in discussions on what needs to be done to address nutrition issues?<br>1. Strongly Agree<br>2. Agree<br>3. Disagree<br>4. Strongly disagree<br>98. Don’t know
13.	Was there a demand/suggestion from other ministries/sectors for your office to collaborate with them on nutrition related issues?<br>1. Strongly Agree<br>2. Agree<br>3. Disagree<br>4. Strongly disagree

	98. Don't know<br>99. Not applicable
14.	Do you feel that you effectively collaborated with your co-workers to address nutrition problems of this sector?<br>1. Strongly Agree<br>2. Agree<br>3. Disagree<br>4. Strongly disagree<br>98. Don't know<br>99. Not applicable
15.	Do you feel that you personally have sufficient access to budgetary resources to be effective in your own responsibilities?<br>1. Strongly Agree<br>2. Agree<br>3. Disagree<br>4. Strongly disagree<br>98. Don't know<br>99. Not applicable
16.	Do you feel that your office/department has sufficient financial resources (budget) to implement the necessary actions to meet its responsibilities?<br>1. Strongly Agree<br>2. Agree<br>3. Disagree<br>4. Strongly disagree<br>98. Don't know
17.	Do you feel that there is a champion for nutrition in your sector? (ie, someone strong making the case for more attention and resources for nutrition action)<br>1. Strongly Agree<br>2. Agree<br>3. Disagree<br>4. Strongly disagree<br>98. Don't know

18.	Is there clear leadership on nutrition in your sector (in terms of supervisors and managers clearly defining what needs to be done for nutrition)?<br>1. Strongly Agree<br>2. Agree<br>3. Disagree<br>4. Strongly disagree<br>98. Don't know
19.	Do you feel that you personally have been adequately trained to carry out your responsibilities?<br>1. Strongly Agree<br>2. Agree<br>3. Disagree<br>4. Strongly disagree<br>98. Don't know
20.	Do you feel that your own colleagues have the right skills/training to be effective in their work?<br>1. Strongly Agree<br>2. Agree<br>3. Disagree<br>4. Strongly disagree<br>98. Don't know
21.	Do you feel that you know how to obtain any technical support for the activities under your responsibility (if you needed to)?<br>1. Strongly Agree<br>2. Agree<br>3. Disagree<br>4. Strongly disagree<br>98. Don't know
22.	Do you feel that you have adequate support from your supervisors/manager for implementing your roles and responsibilities?<br>1. Strongly Agree<br>2. Agree<br>3. Disagree<br>4. Strongly disagree<br>98. Don't know

23.	Do you feel that you have adequate commitment from colleagues in your sector to help you fulfill your roles and responsibilities?<br>1. Strongly Agree<br>2. Agree<br>3. Disagree<br>4. Strongly disagree<br>98. Don’t know
24.	Do you feel that sufficient non-financial resources (office supplies) that facilitate you in carrying out your responsibilities are made available your office/department?<br>1. Strongly Agree<br>2. Agree<br>3. Disagree<br>4. Strongly disagree<br>98. Don’t know
